# The Effect of Caffeine Ingestion and Carbohydrate Mouth Rinse on High-Intensity Running Performance

**DOI:** 10.3390/sports7030063

**Published:** 2019-03-14

**Authors:** Mark Germaine, Kieran Collins, Marcus Shortall

**Affiliations:** Center for Exercise and Metabolic Science, Department of Applied Science, Technological University—Dublin, Dublin 24, Ireland; kieran.collins@ittdublin.ie (K.C.); marcus.shortall@irfu.ie (M.S.)

**Keywords:** dietary supplement, energy, nutraceutical, nutrition, nutrient, sports performance

## Abstract

The aim of the current study was to investigate whether carbohydrate mouth rinsing works synergistically with caffeine to augment high-intensity running in a fed state. Eight participants completed a total of three trials; (1) placebo (PLA) trial (placebo capsule + placebo mouth rinse), (2) caffeine (CAF) trial (400 mg caffeine + placebo mouth rinse) and (3) carbohydrate mouth rinse + caffeine (CMR + CAF) trial (400 mg caffeine + 6% carbohydrate mouth rinse). Each trial consisted of a 45 min steady-state run at 65% VO_2max_, followed by 90% VO_2max_ high-intensity intervals (HIIT) at 1 min and subsequently by a 1 min recovery walking at 6 km·h^−1^, until exhaustion. Both CAF (46.8 ± 20.1 min) and CMR + CAF (46.9 ± 18.4 min) time to exhaustion were significantly greater than the PLA group (36.2 ± 14.8 min, *p* < 0.001). Post hoc analysis revealed that there was a significant increase in time to exhaustion between PLA and CMR + CAF (*p* = 0.006) and PLA and CAF (*p* = 0.017) but not between CAF and CMR + CAF (*p* = 0.99). In conclusion, we provide novel data that suggest that caffeine alone would likely suffice as an ergogenic aid during high-intensity running while in a fed state.

## 1. Introduction

It has been hypothesized that the benefits of exogenous carbohydrate during high-intensity exercise may be mediated through the activation of carbohydrate taste receptors in the mouth, which subsequently activate the central nervous system (CNS) [1]. Carter et al. [2] had participants rinsing a 6.4% maltodextrin solution around their mouth for 5 s before spitting it out each 12.5% of the trial, which was a 40 km cycle time trial (every 5 km). In comparison to a taste-matched placebo, the carbohydrate mouth rinse (CMR) trial was completed 2.9% faster. Later, Chambers et al. [1] examined the effects of glucose, maltodextrin and artificial sweeteners on exercise performance and by functional magnetic resonance imaging (*f*MRI). The authors demonstrated comparable performance improvements with both maltodextrin and glucose, corresponding to 3.1% and 2%, respectively, and also demonstrated that both substrates activated reward-related regions of the brain, while artificial sweeteners failed to have the same effect. These findings would lend support to the hypothesis of CNS-mediated effects of CMR which may be independent of taste or sweetness, given that maltodextrin is a flavourless polymer of glucose and that artificial sweeteners mimicking a sweet taste failed to provide the same response.

Whitham and McKinney [3] were the first to follow up Carter et al. but utilised running as opposed to cycling. Surprisingly, the results of this investigation contrasted with those of the mentioned study, and no performance benefit from CMR was observed. Similarly, Beelan et al. [4] attempted to perform a follow-up and repeat the study design of Carter et al. Once again, the authors observed no measurable benefit in performance from CMR. The reason for the contrast in results may be the pre-exercise meal that was consumed 4 h prior [3] and 2 h prior to exercise [4], which may negate some of the performance benefits associated with carbohydrate mouth rinsing.

Fares and Kayser [5] designed a study to directly examine the difference in the ergogenic effect of mouth rinsing in fasted and fed states. The results from this investigation demonstrated significant performance benefits for both conditions; however, mouth rinsing when in the fasted state resulted in an 11.6% increase in performance compared to 3.5% measured in the fed condition, with respect to a placebo. More recently, Lane et al. [6] reproduced similar findings in highly trained athletes; however, the magnitude of performance benefit was less pronounced (fed 3.3% vs fasted 1.8%). The findings of these performance investigations lend support to the previous work of Haase et al. [7] who examined the activation of various regions of the brain using *f*MRI in both fasted and fed states. The investigation demonstrated that brain activity was significantly greater in some areas of the brain when sucrose was consumed in the fasted state than in the fed state. Thus, it would appear that the benefits of carbohydrate mouth rinsing may be more pronounced in a fasted state. However, evidence still suggests it is an effective fueling strategy even when used in a fed state.

Kasper et al. [8] investigated the addition of caffeine ingestion to CMR during a high-intensity running protocol. The authors of this study found that adding caffeine ingestion to the mouth rinse during a carbohydrate-restricted state enhanced the ergogenic effect seen with carbohydrate mouth rinsing. More recently, Devenney et al. [9] performed a follow-up to the study of Kasper et al. [8], investigating the effects of caffeine ingestion and mouth rinse in the fed state. The authors once again found significantly greater increases in performance with the addition of caffeine ingestion compared to a placebo. Caffeine primarily acts on the central nervous system as an adenosine receptor antagonist [10], thus preventing tiredness and, as a result, prolonging the time to fatigue. This poses the question of whether a synergistic effect on the central nervous system occurred or whether caffeine alone could exert this effect, given the robust evidence demonstrating the ergogenic effects of caffeine supplementation on endurance exercise [11].

Therefore, the aim of the present study was to investigate whether carbohydrate mouth rinsing works synergistically with caffeine to augment high-intensity running performance in recreationally trained males in a carbohydrate-fed state, compared to caffeine only or a placebo. We hypothesized that the addition of a carbohydrate mouth rinse would not promote a greater performance benefit compared to caffeine alone when the participants are in a fed state.

## 2. Materials and Methods

### 2.1. Experimental Design

**Figure 1:**
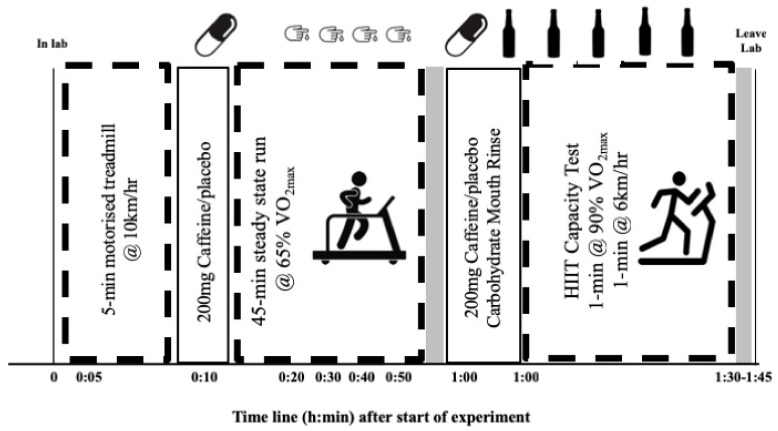
Schematic of experimental design and protocol upon lab visit and time to completion. VO_2max_, maximal oxygen uptake; HIIT, high-intensity intervals.

The current investigation was a randomized, repeated-measure and double-blind trial in which participants reported for laboratory-based testing a total of four times. Upon the participants first visit, height and body mass were recorded prior to commencing an incrementally graded treadmill test to assess maximal oxygen uptake (VO_2max_). During the three experimental trials, participants undertook a steady-state running exercise protocol (45 min at 65% VO_2max_), followed by a high-intensity interval training (HIIT) protocol to exhaustion (1 min bouts at 90% VO_2max_ interspersed with 1 min bouts walking at 6 km·h^−1^). Participants received 200 mg caffeine (BulkPowders, Essex, UK) or 200 mg whey protein (BulkPowders, Essex, UK) placebo in gel capsule format (Size 0 gelatin capsules; BulkPowders, Essex, UK), immediately prior to commencing the steady-state exercise protocol and again immediately prior to commencing the HIIT protocol. Further to this, during the HIIT protocol, participants received a 6% maltodextrin mouth rinse or a 0% taste-matched placebo at 4 min intervals, which they were required to rinse around their mouth for 5 seconds before spitting into a waste bucket, as per Devenney et al. [8]. Therefore, participants completed three exercise trial conditions: a placebo (whey placebo capsule and CMR placebo), a caffeine trial (CAF) and a carbohydrate mouth rinse with caffeine trial (CMR + CAF). The primary outcome measured was the time ran at the end of the HIT to exhaustion. An overview of the experimental design is presented in Figure 1.

### 2.2. Participants

Eight recreationally trained males (age, 23.6 ± 3.9 years; height, 176.1 ± 7.4 cm; mass, 78.1 ± 11.8 kg; VO_2max_, 54.0 ± 6.1 mL·kg^−1^·min^−1^) volunteered to participate in the current investigation. Recreationally trained was defined as having a minimum of two years participating weekly in either endurance running or a field-based game such as football or gaelic football. The details of the experimental protocol were explained to all participants who gave verbal and written consent. Prior to the commencement of testing, the study was approved by the local institution’s ethics committee, the Institute of Technology Tallaght.

### 2.3. Assessment of Anthropometrics and Maximal Oxygen Uptake

At least seven days prior to commencing the experimental protocol, participants reported to the laboratory to undertake anthropometric testing and an assessment of maximal oxygen uptake. Height was measured using a stadiometer (Seca 213, Leicester, UK) to the nearest 0.1 cm utilising the ISAK protocol, and body mass was recorded to the nearest 0.1 kg [12]. Participants performed a continuous incremental treadmill test to exhaustion to obtain the VO_2max_ (Cosmed t170, Rome, Italy). The treadmill gradient was set at a constant 1% to match the energetic cost of outdoor running [13]. The incremental test commenced at a treadmill speed of 6 km·h^−1^ for 4 min followed by a 1 min rest interval before being ramped up 2 km·h^−1^, similar to the protocol used by Akubat et al. [14]. The ramp protocol continued until the participant reached a blood lactate of 4.0 mmol·L^−1^. The treadmill speed was then increased by 1 km·h^−1^ every minute until the participant reached volitional exhaustion. Breath-by-breath analysis was assessed by a quark gas analyser (Quark CPET, Cosmed, Rome, Italy), heart rate was measured with a T31 heart rate monitor (Polar Electro, Espoo, Finland) and lactate was measured with a lactate plus meter (Nova Biomedical, Waltham, MA, USA). VO_2max_ was determined once the following criteria were achieved: (1) heart rate ± 5 b·min^−1^ of age-predicted maximum, (2) RER > 1.1 and (3) 30 s before and after the VO_2_ peak, that is, If criteria (1) and (2) had been met during the test, VO_2max_ data were taken as an average of data points from 30 s prior to the peak and 30 s post-peak VO_2_. The speed at which VO_2max_ was achieved was used to prescribe the running speeds for the steady-state and HIIT protocols [15].

### 2.4. Steady-State Exercise and HIIT Running Protocol

Upon arrival to the laboratory, participants immediately consumed either a 200 mg caffeine capsule or a 200 mg placebo capsule before commencing the 45 min steady-state protocol, allowing for peak plasma levels of caffeine to occur upon cessation of the steady-state and prior to commencing the HIIT protocol [16]. The steady-state running protocol consisted of a continuous 45 min run at 65% of VO_2max_ at a 1% gradient [13]. Lactate response was measured at the 10, 20, 30 and 40 min mark to ensure blood lactate remained below 4.0 mmol·L^−1^. Following the steady-state protocol, participants consumed another capsule of caffeine or placebo and began the HIIT protocol as per Kasper et al. [8] and Devenney et al. [9]. The HIIT protocol was an adapted version of the one by Kasper et al. [8] which consisted of 1 min bouts of running at 90% VO_2max_ interspersed with 1 min recovery periods at 6 km·h^−1^ until exhaustion, and similar to that of Devenney et al. [9]. During the HIIT protocol, participants rinsed 25 mL of a 6% carbohydrate solution or a taste-matched placebo for periods of 5 s every 4 min or after every second HIIT bout [8]. This continued for the duration of the HIIT protocol, which ceased once the participant was exhausted and could no longer continue in the trial. After completion of the HIIT protocol, the time taken to reach exhaustion was recorded.

### 2.5. Dietary Control and Blinding

Participants were provided with a sample carbohydrate-loading protocol to use as a guideline for consumption on the day before the experimental trials. The day prior to testing, the participants were reminded to eat a carbohydrate-rich diet in an attempt to consume 6 g·kg^−1^ of bodyweight (bw) carbohydrate, as per the dietary template provided. This dietary template was further broken down into diets for weight categories. Participants were also instructed to eat a carbohydrate-rich breakfast 2–3 h prior to arriving to the lab, providing 2 g·kg^−1^·bw^−1^ carbohydrate, and were asked to repeat this breakfast, in addition to replicating the previous days diet, for each of the three experimental trials. In this way, participants consumed the identical diet prior to each of the three experimental trials.

Caffeine anhydrous powder, whey protein powder (vanilla) and size 0 gelatin capsules (BulkPowders, Essex, UK) were used in the preparation of the placebo and caffeine. The placebo mouth rinsing solution was prepared with the use of water and diluted sugar-free orange juice (Robinson Squash, Britvic Orange Soft Drinks, Hertfordshire, UK), while the carbohydrate mouth-rinsing solution was prepared with the addition of maltodextrin powder (BulkPowders, Essex, UK) to the sugar-free juice, to ensure the solutions were identical in colour and taste.

### 2.6. Statistical Analysis

A priori test using the effect sizes seen in Kasper et al. and Devenney et al. suggested that we would need, to be conservative, 12 participants, or if liberal, 6 participants. On the basis of both previous papers using eight participants and finding significant results, eight participants were selected as the sample size. A one-way repeated measures ANOVA was conducted to determine whether there was a statistically significant difference in the total time during the high-intensity protocol between PLA, CAF and CMR + CAF groups (version 18 for Windows, SPSS Inc, Chicago, IL, USA). Bonferroni adjustment was used during post hoc analysis if a significant effect was observed. Prior to analysis, all data were analysed for normal distribution using the Shapiro–Wilks test and box plots for outliers. Cohens *d* effect size was calculated between conditions, where *d* = 0.2 is considered a “small” effect size, 0.5 represents a “moderate” effect size and 0.8 a “large” effect size. Differences between conditions for heart rate, lactate and rating of perceived exertion (RPE) were evaluated using a 3 × 6 general linear model (GLM) with repeated measures during the steady-state trial. Differences for heart rate, lactate and RPE between the steady-state running and HIIT running were analysed with a paired *t*-test, taking the final stage during the steady-state run. All data are reported as means ± standard deviation unless stated otherwise, and 95% confidence intervals are reported where appropriate. Significance was set at *p* < 0.05.

## 3. Results

### 3.1. Exercise Capacity during HIIT

**Figure 2:**
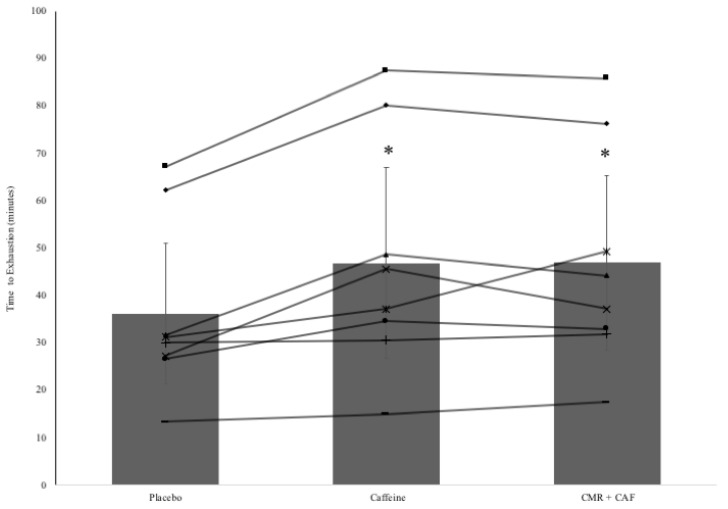
Group and individual participant mean ± standard deviation of time to exhaustion during the HIIT protocol (y-axis) with placebo (36.2 ± 14.8 min), caffeine (46.8 ± 20.1 min) and carbohydrate mouth rinse + caffeine (CMR + CAF; 46.9 ± 18.4 min); * denotes significantly greater than placebo.

**Figure 3:**
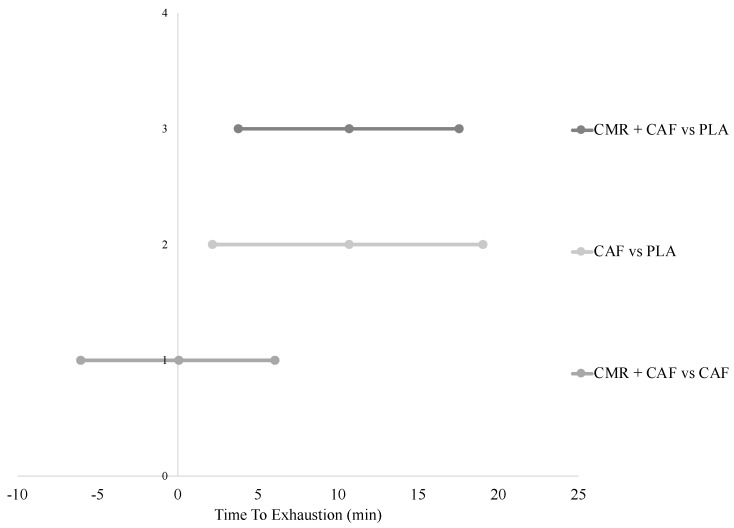
Forest plot of the mean difference between conditions with upper and lower bound 95% confidence intervals. Level 3 (y-axis) represents the difference between carbohydrate mouth rinse + caffeine and placebo (almost certainly beneficial). Level 2 represents the difference between caffeine only and placebo (PLA, almost certainly beneficial). Level 1 represents the difference between carbohydrate mouth rinse + caffeine and caffeine only (unclear).

The results of the HIIT exercise test to exhaustion can be seen in Figure 2, with both group and individual changes. There was a significant effect of the supplement on the time to exhaustion during the HIIT test (F = 14.345; *p* < 0.001; η^2^ = 0.672; 1 − β = 99.3%). Post hoc analysis using the Bonferroni adjustment revealed that there was a significant increase in time to exhaustion between PLA and CMR + CAF (*p* = 0.006; *d* = 0.51; moderate), with a mean increase of 10.7 min (29%) (95% CI = 3.8 to 17.6, Figure 3), and between PLA and CAF (*p* = 0.017; *d* = 0.49; moderate), with a mean increase of 10.7 min (29%) (95% CI = 2.2 to 19.1, Figure 3), but not between CAF and CMR + CAF (*p* = 0.99; *d* = 0.01; small), with a mean difference of −0.1 min (95% CI = −6 to 6.1, Figure 3).

### 3.2. Physiological Responses during Steady State and HIIT

There was no significant effect of condition on lactate (*p* = 0.253), RPE (*p* = 0.691) or heart rate (*p* = 0.466) responses; however, heart rate (*p* < 0.001) and RPE (*p* < 0.001) significantly rose throughout the steady-state protocol and again after exhaustion (*p* < 0.001). While lactate did not rise significantly during steady (*p* = 0.191), it was significantly higher after exhaustion (*p* < 0.0005). There was no interaction effect observed for lactate (*p* = 0.177), RPE (*p* = 0.524) or heart rate (*p* = 0.225). Lactate, RPE and heart rate responses can be seen in Table 1.

## 4. Discussion

The current investigation is one of the first to begin to understand the potential effects of co-supplementation in recreational endurance athletes. As such, we aimed to investigate whether CMR works synergistically with caffeine to augment high-intensity running performance when compared to caffeine alone or a placebo in a carbohydrate-fed state in recreationally trained males. The main findings of this investigation would suggest that, while there may be a benefit to CMR and caffeine ingestion on high-intensity running performance (+29% time to exhaustion), there does not appear to be any additional benefit from the addition of CMR to caffeine ingestion. Our current data suggest that the ergogenic effect of caffeine is moderate in nature when contrasted with the small effect of the addition of CMR to caffeine. This may be in part due to the carbohydrate-loading and fed state of the participants, mitigating any potential additive effects of the CMR [6].

The addition of CMR to caffeine within the current investigation resulted in no meaningfully observable effect (*p* = 0.99, *d* = 0.01). In comparison, Kasper et al. [8] ran a protocol similar to the present study, except for the fact that it was run in a carbohydrate-restricted state; Devenney et al. [9] later followed up this study, considering a carbohydrate-fed state. The present study followed up these two previous studies, except that this time, a caffeine control arm was included, in the attempt to draw comparable results across the three studies. In both previous studies mentioned, while both the CMR (+44% Kasper et al.; +13% Devenney et al.) and CMR + CAF (+78% Kasper et al.; +41% Devenney et al.) groups improved the time/distance to exhaustion, there appeared to be a notable further improvement with the addition of caffeine to the CMR. The main difference between these two studies was the magnitude of performance improvement in the CMR group, with it being more pronounced in the fasted condition [8]. Taken together, this data suggest that there does not appear to be any benefit from co-ingesting CMR and caffeine when exercising in a fed state (Figure 3). In addition to the feeding state, a caffeine-dosing strategy may also reduce the effectiveness of CMR, as the double dosing of 200 mg caffeine would result in a cumulative increase in plasma caffeine levels [16].

Our findings lend support to an ever-growing thought according to which co-supplementation of multiple ingredients may not exactly confer greater benefit than isolated supplementation [17]. For example, Mero et al. [18] demonstrated that, while sodium bicarbonate significantly reduced the time taken to complete repeated maximal swims compared to placebo, there was no additional benefit from co-ingestion of beta-alanine, another supplement which acts as a buffer, similar to sodium bicarbonate. Similarly, while both caffeine and CMR work to stimulate the CNS, they do so through two separate mechanisms. A case may therefore be made, following the results of the current study, that no additional benefit of carbohydrate mouth rinse may be obtained through co-supplementation in a fed state.

Christensen et al. [19] demonstrated that caffeine supplementation alone and in combination with sodium bicarbonate significantly increased 6 min maximal performance in 12 rowers; however, there was no difference compared with the co-ingestion of sodium bicarbonate. Similarly, Kilding et al. [20] found no further increase in high-intensity cycling performance when sodium bicarbonate was used in combination with caffeine. However, Felippe et al. [21] examined the use of sodium bicarbonate and caffeine co-ingestion in judokas, and while individually there may have been some benefit from each supplement, the combination of supplements significantly increased judo throws during a specialized test. This may suggest that the ergogenic effects of caffeine are possibly potent enough to offset any ergogenic effects of additional nutritional or supplemental aids.

Our study is one of the first to differentiate the effects of co-ingesting CMR and caffeine. The current area of co-supplementation is significantly under-researched, yet it is common practice for athletes and recreational athletes to consume multiple supplements, highlighting the ecological validity of the current investigation. In agreement with this, common ingredient lists of “pre-workout” formulas often contain a blend of various ingredients, most notably caffeine, with no evidence, however, supporting the efficacy of these blends. In fact, some evidence suggests that caffeine may be providing most of the ergogenic benefits [19]. We would therefore suggest that any supplement which contains caffeine be tested not only against a placebo but also against a dose-matched caffeine arm, to ensure efficacy of the supplement and differentiate its effects from those of caffeine alone.

### Limitations

During the exercise trials, participants rinsed a 6% maltodextrin solution around their mouth for a total of 5 s before spitting into a bucket. Sinclair et al. [22] examined the effects rinse duration had on cycling performance and found that a 5 s rinse improved performance by 4.7%, but these findings were statistically non-significant. In contrast, rinsing for 10 s produced a statistically significant 6.3% increase in performance. Further to this, Rollo et al. [23] demonstrated that ingesting a carbohydrate solution following a 5 s rinse resulted in significantly greater running distances during a 1 h run compared to rinsing the mouth with the solution and spitting. Taken together, this may suggest that rinsing with the solution for up to 10 s followed by ingestion of the fluid may provide a better CMR strategy.

Also, it is worth noting that the results of this investigation only focused on one primary physiological variable, i.e., time to exhaustion, so we cannot rule out the possible synergistic effect of these supplements on other physiological metrics such as power, oxygen kinetics, internal stress or decision-making. Also, it was our intention to implement to 45 min steady-state exercise protocol to try garner comparisons to previous research [8,9]. However, it may be appropriate to eliminate this steady-state exercise protocol and simply test high-intensity running performance, as glycogen depletion is not a goal when exercising in the fed state.

Finally, while participants were instructed to consume a high-carbohydrate diet as per the instructions, it may be possible that some participants did not adhere to these recommendations. If some participants lied about consuming the diet, this would result in differing muscle glycogen levels and could consequently effect the time to fatigue during the HIIT running. Further to this, while there is some debate about the relevancy of habitual caffeine consumption [24,25,26,27], habitual caffeine consumers were not excluded from this study, and this may have affected participants’ responsiveness to caffeine supplementation.

## 5. Conclusions

To conclude, the addition of a CMR to caffeine and the process of co-supplementation may not work synergistically to further enhance performance during an exercise capacity test. This may mitigate any need to perform a mouth rinse within a competition scenario, which can be difficult to implement within specific sporting scenarios that do not allow for consistent breaks in play. Thus, in a competitive scenario, the application of a CMR may be limited to events when caffeine consumption is not a feasible option, such as those undertaken late in the evening or with athletes who do not consume caffeine. However, it remains unknown whether CMR may exert an effect when co-ingested with caffeine in the fasted state, something which may be considered for future research. Further to this, much of the research of co-ingestion of supplements thus far has been limited to sodium bicarbonate, and as such, it may be useful for further research to examine the potential effects of co-ingesting other supplements, with a particular focus on caffeine, as it is the most widely consumed drug/supplement on the market.

## Figures and Tables

**Table 1:** Physiological mean responses of heart rate, blood lactate and rating of perceived exertion (RPE) during steady-state running and after exhaustion. No statistical differences were observed between groups, but there were significant effects for time during the steady-state run for heart rate and RPE (*p* < 0.05) and at the completion of HIIT for heart rate, lactate and RPE (*p* < 0.05).

Measure		5	10	20	30	40	45	Exh
Heart Rate	Placebo	153 ± 12	161 ± 10	167 ± 14	170 ± 15	170 ± 14	171 ± 15*	190 ± 6*
	CHO	152 ± 10	160 ± 9	165 ± 15	167 ± 10	170 ± 11	172 ± 12*	189 ± 6*
	CHO + Caff	150 ± 10	158 ± 12	165 ± 11	170 ± 9	173 ± 12	174 ± 16*	191 ± 17*
Lactate	Placebo	2.5 ± 1.4	2.3 ± 1.1	2.6 ± 1.2	2.6 ± 1.3	2.5 ± 1.4	2.4 ± 1.3	10.3 ± 2.8*
	CHO	1.9 ± 0.9	2.2 ± 1.1	2.0 ± 0.9	2.0 ± 1.0	2.0 ± 1.1	2.2 ± 1.0	10.4 ± 2.3*
	CHO + Caff	1.9 ± 1.1	2.1 ± 0.7	2.3 ± 1.2	2.6 ± 1.2	3.0 ± 1.5	2.5 ± 1.4	10.8 ± 3.0*
RPE	Placebo	10 ± 2	10 ± 3	11 ± 2	13 ± 2	14 ± 3	14 ± 3*	20 ± 1*
	CHO	8 ± 2	10 ± 2	12 ± 2	14 ± 3	14 ± 2	14 ± 3*	20 ± 1*
	CHO + Caff	9 ± 3	11 ± 2	11 ± 3	13 ± 1	13 ± 2	14 ± 2*	20 ± 1*

* indicates significant effect of time; CHO = carbohydrate mouth rinse; CHP + Caff = carbohydrate mouth rinse with caffeine ingestion; RPE = rating of perceived exertion (Borg Scale).
